# Buffalo Law School—Law Department of Niagara University

**Published:** 1887-03

**Authors:** 


### Editorial.

### *BUFFALO LAW SCHOOL.*

### LAW DEPARTMENT OF NIAGARA UNIVERSITY.

We are called upon again to record another addition to the educational advantages of Buffalo, and to congratulate the Niagara University on its new acquisition.

When this enterprising and progressive institution erected a medical school in Buffalo, we were informed by one in authority and in whom we all have confidence, that other departments would also be established here, and additions made to an already powerful institution. To-day that promise is made good.

As is well known, this institution has a collegiate department where young men are instructed in the arts and sciences, and where, after a strict examination, the student can obtain any degree conferred by such colleges. In addition to these, it has a theological department. They are conducted at Suspension Bridge, where are located commodious buildings. The medical department, founded four years ago in Buffalo, has been in every way a success. The faculty has kept its promises to the profession and the public to maintain a school on a higher plane. It has constantly insisted on preliminary requirements and a thorough medical education before graduation. By such a course, and by the effort of the members of the faculty to merit the honor given them, they have won the respect and confidence of the medical fraternity. Thus it is that success in one direction is apt to be followed by achievements in other fields. The legal profession has united and joined hands in furthering these ideas of higher education. It is conceded that a law school is quite necessary to the student, and yet the majority of the lawyers of our city have never attended a school to learn their chosen profession. By the organization of this department, an opportunity will be afforded many to partake of the advantages of such an institution who are not able or willing to take a course away from home. The gentlemen who have undertaken this task are men of ability and energy. They will, as they say, endeavor to maintain a school second to none in this country. They have been unusually fortunate in the selection of a man who can devote a great deal of time to the work of the school, one who not only has the time but the education, experience and ability to perform this work. As to his co-laborers, quite as much can be said; they are eminently qualified in the various departments they will undertake; of the assistants, which have thus far been selected, it can be truly said that they are young men of excellent education and attainments, and doubtless by this early experience will be, in later life, able to take a superior place.

The Faculty of the Medical Department has assigned rooms to the new school for the present. Additions to the faculty, and the various topics, will be announced in the near future. The legal profession has received the announcement of the school with great enthusiasm. The daily press have echoed the same sentiments. We append these, as follows:

[From the Buffalo Commercial.]

The organization of a law school in Buffalo, under the charter of the Niagara University, is an event of decided local interest. Such an extension of local educational facilities has been discussed often enough, and the time now seems ripe for the execution of the project. The sponsors of the movement are Spencer Clinton, the Hon. James Sheldon, the Hon. George Clinton, Adelbert Moot, Sheldon T. Viele, E. Corning Townsend and Charles P. Norton. The names of these gentlemen are a sufficient guarantee of the character and aims of the proposed school. They are men of talent, energy and experience in the legal profession of Buffalo. With their influence, and with the organization of a competent faculty under their direction, there appears to be no reason why the Buffalo Law School should not succeed. The older and famous law schools are none of them very near to Western New York, and to take a course of study at any of them involves an expense that closes them against many poor and worthy young men. The new Buffalo school will hold out strong inducements to those who desire to study law in a city where board is cheap.

[From the Buffalo Courier.]

Some years ago there was talk of the establishment of a law department in the University of Buffalo. No active steps were taken, however, and the project was allowed to go by default. The charter of the Niagara University provides for a law department, and the question has been under advisement quietly for some time. The matter has now taken practical form, and is about to become a fact. It is to be known as the Buffalo Law School, and is created as a department of the Niagara University. The school will be under the direction of the following able gentlemen, who have initiated the movement, and who stand in the foremost rank of the legal profession: Spencer Clinton, the Hon. James Sheldon, the Hon. George Clinton,

Adelbert Moot, Sheldon T. Viele, E. Corning Townsend and Charles P. Norton.

The step was taken after mature and deliberate reflection and with a view of promoting the interests of professional learning, and advancing the intellectual as well as the material enterprise of Buffalo. That such a movement as this is timely, is evident to all those who realize the large growth to which Buffalo has attained, and the large number of young men in the city and within the extended surrounding territory tributary to it, who need and demand thorough and systematic training for the profession into which they are to enter. That such a school will succeed is guaranteed by the fact that many of the best lawyers have approved and commended its organization, and have expressed the belief that it will have hearty support of the bar generally, and have signified their willingness to aid it in every possible way.

Heretofore, the course has not seemed clear for the organization of the much-desired school, but now, with the inducements and facilities offered by this rapidly-growing and powerful institution of learning, an institution which, in its several departments of arts, sciences, etc., is honoring the educational interests of the Empire State, the way appears to be open, and the opportunity has been improved. The Medical Department of the Niagara University, now in its fourth year, has already distinguished itself as a most worthy institution, and in its efforts to make better physicians, has commanded the respect and received the praise of Buffalo's best citizens. The same commendable efforts which will be put forth by the Law department to advance legal learning and culture, will, without doubt, be appreciated by the people of Buffalo, and they will be proud of such an institution. The Law School will begin in the fall, and for a while will be maintained at the Medical College building, on Ellicott street, where excellent rooms have been tendered for that purpose.

[From the Buffalo Express.]

The long-talked-of Law School is a mere matter of talk no longer. Steps have been taken to establish a Law School in connection with the Niagara University, the medical department of which is situated on Ellicott street, near Broadway. The project has been more or less actively canvassed for a number of years, but nothing came of it until within a few days, when it was intimated by the authorities of the Niagara University that they would be willing to furnish the necessary accommodations for law lectures, if it was thought desirable to establish a school. Accordingly, a meeting was held on Thursday by a number of prominent lawyers, and what may be called a preliminary organization effected.

The gentlemen connected with the enterprise are ex-Judge Sheldon, the Hon. George Clinton, Spencer Clinton, Adelbert Moot, Sheldon T. Viele, E. Corning Townsend and C. P. Norton. So far very little has been done further than to form an agreement to go forward with the work, and to send a petition to the University, asking the privilege of establishing the school.

One point, as stated by one of the gentlemen interested, is definitely arranged. Ex-Judge Sheldon has been prevailed on to take the laboring oar. He will give his whole time to the work, and will, at present at least, be the only one who will do this. The other lawyers mentioned will take professorships and deliver courses of lectures, but they can do this without giving up their practice. The gentlemen consider that the position of ex-Judge Sheldon, he having just retired from the bench, and his legal and literary abilities, specially fit him for the work. He will take the professorship of municipal law. Beyond this no positions have been arranged, though it is understood that Messrs. Spencer Clinton, George Clinton, Moot and Viele will accept the remaining four of the five full professorships usually connected with a law school, and Messrs. Townsend and Norton will take assistant professorships. The college will start up next fall.

---

It is with pleasure that we announce the return of Dr. Herman Mynter from Europe, where he has passed several months in the hospitals for study and observation. With health fully restored, he proposes to engage in the practice of his profession, bringing renewed zeal to his works and the added experience and maturity of judgment secured through his travels abroad. We feel the deepest interest in the success of our late editorial conferree, and bespeak for him the earnest co-operation of the profession and the support of the public, which in the past he has done so much to merit.

